# Bovine milk in human nutrition – a review

**DOI:** 10.1186/1476-511X-6-25

**Published:** 2007-09-25

**Authors:** Anna Haug, Arne T Høstmark, Odd M Harstad

**Affiliations:** 1 Department of Animal and Aquacultural Sciences, Norwegian University of Life Sciences, Aas, Norway; 2 Section of Preventive Medicine and Epidemiology, University of Oslo, Oslo, Norway

## Abstract

Milk and milk products are nutritious food items containing numerous essential nutrients, but in the western societies the consumption of milk has decreased partly due to claimed negative health effects. The content of oleic acid, conjugated linoleic acid, omega-3 fatty acids, short- and medium chain fatty acids, vitamins, minerals and bioactive compounds may promote positive health effects. Full-fat milk has been shown to increase the mean gastric emptying time compared to half-skimmed milk, thereby increasing the gastrointestinal transit time. Also the low pH in fermented milk may delay the gastric emptying. Hence, it may be suggested that ingesting full-fat milk or fermented milk might be favourable for glycaemic (and appetite?) regulation. For some persons milk proteins, fat and milk sugar may be of health concern. The interaction between carbohydrates (both natural milk sugar and added sugar) and protein in milk exposed to heat may give products, whose effects on health should be further studied, and the increasing use of sweetened milk products should be questioned. The concentration in milk of several nutrients can be manipulated through feeding regimes. There is no evidence that moderate intake of milk fat gives increased risk of diseases.

## Introduction

Bovine milk and dairy products have long traditions in human nutrition. The significance of milk is reflected in our northern mythology where a cow named Audhumla was evolved from the melting ice. She had horn and milk was running as rivers from her teats. This milk was the food for Ymer, the first creature ever existing [1].

The consumption of milk and milk products vary considerably among regions; of drinking milk from about 180 kg yearly per capita in Island and Finland to less than 50 kg in Japan and China [2]. In the western societies, the consumption of milk has decreased during the last decades [3]. This trend may partly be explained by the claimed negative health effects that have been attributed to milk and milk products. This criticism has arisen especially because milk fat contains a high fraction of saturated fatty acids assumed to contribute to heart diseases, weight gain and obesity [4].

The association between food and health is well established [4] and recent studies have shown that modifiable risk factors seem to be of greater significance for health than previously anticipated [5]. Prevention of disease may in the future be just as important as treatment of diseases. Indeed, many consumers of today are highly aware of health-properties of food, and the market for healthy food and food with special health benefits is increasing.

Milk is a complex food made up of components, which per se may have negative or positive health effects, respectively. Milk composition can be altered by the feeding regime. The main aim of this review is to discuss effects of milk components that are of particular interest for human health, and to give an overview of the potential for manipulation of bovine milk by feeding regimes to the lactating cows, thus giving improved nutritional composition of the milk for human consumption.

## Milk composition in general

Bovine milk contains the nutrients needed for growth and development of the calf, and is a resource of lipids, proteins, amino acids, vitamins and minerals. It contains immunoglobulins, hormones, growth factors, cytokines, nucleotides, peptides, polyamines, enzymes and other bioactive peptides. The lipids in milk are emulsified in globules coated with membranes. The proteins are in colloidal dispersions as micelles. The casein micelles occur as colloidal complexes of protein and salts, primarily calcium [6]. Lactose and most minerals are in solution. Milk composition has a dynamic nature, and the composition varies with stage of lactation, age, breed, nutrition, energy balance and health status of the udder. Colostrums differ considerably to milk; the most significant difference is the concentration of milk protein that may be about the double in colostrum compared to later in lactation [7]. The change in milk composition during the whole lactation period seems to match the changing need of the growing infant, giving different amounts of components important for nutrient supply, specific and non-specific host defence, growth and development. Specific milk proteins are involved in the early development of immune response, whereas others take part in the non-immunological defence (e.g. lactoferrin). Milk contains many different types of fatty acids [8]. All these components make milk a nutrient rich food item.

## Components in milk and their health effects

### Lipids

#### Fatty acids

In average, milk contains about 33 g total lipid (fat)/l [9] (Table 1). Triacylglycerols, which account for about 95 % of the lipid fraction, are composed of fatty acids of different length (4–24 C-atoms) and saturation [8]. Each triacylglycerol molecule is built with a fatty acid combination giving the molecule liquid form at body temperature. Other milk lipids are diacylglycerol (about 2% of the lipid fraction), cholesterol (less than 0.5 %), phospholipids (about 1%), and free fatty acids (FFA) accounting to less than 0.5% of total milk lipids [8]. Increased levels of FFA in milk might result in off-flavours in milk and dairy products, and the free volatile short-chain fatty acids contribute to the characteristic flavours of ripened cheese.

**Table 1:** Milk composition and percent contribution to the daily dietary reference intakes of some nutrients in 0.5 l whole milk, and their main health effects

**Milk component**	**Concentration in 1 l whole milk**^a^	**Percent contribution of 0.5 l whole milk to reference intake**^b^	**Health effects**
Fat	33 g/l		Energy rich
Saturated fatty acids	19 g/l		Increase HDL, small dense LDL, and total cholesterol. Inhibition of bacteria, virus
Oleic acid	8 g/l		Prevent CHD, gives stable membranes
Lauric acid	0,8 g/l		Antiviral and antibacterial
Myrisitc acid	3,0 g/l		Increase LDL and HDL
Palmitic acid	8 g/l		Increase LDL and HDL
Linoleic acid	1,2 g/l		Omega-6 fatty acid
Alpha linolenic a	0,75 g/l		Omega-3 fatty acid
Protein	32 g/l	30–40%	Essential amino acids, bioactive proteins, peptides. Enhanced bioavailability
Lactose	53 g/l		Lactosylation products
Calcium	1,1 g/l	40–50%	Bones, teeth, blood pressure, weight control
Magnesium	100 mg/l	12–16%	For elderly, asthma treatment
Zinc	4 mg/l	18–25%	Immune function. Gene expression
Selenium	37 ug/l	30%	Cancer, allergy, CHD
Vitamin E	0,6 mg/l	2 %	Antoixidant
Vitamin A	280 ug/l	15–20%	Vision, cell differentiation
Folate	50 ug/l	6 %	DNA synthesis, cell division, amino acid metabolism
Riboflavin	1,83 mg/l	60–80%	Prevent ariboflavinosis
Vitamin B_12_	4,4 ug/l	90%	Key role in folate metabolism

^a ^data from USDA Food Composition Data [9].

^b ^Dietary reference intake (DRI) for men and women [4].

#### Saturated fatty acids

More than half of the milk fatty acids are saturated, accounting to about 19 g/l whole milk [9] (Table 1). The specific health effects of individual fatty acids have been extensively studied [10-13]. Butyric acid (4:0) is a well-known modulator of gene function, and may also play a role in cancer prevention [12]. Caprylic and capric acids (8:0 and 10:0) may have antiviral activities, and caprylic acid has been reported to delay tumour growth [11]. Lauric acid (12:0) may have antiviral and antibacterial functions [14], and might act as an anti caries and anti plaque agent [15]. Interestingly, Helicobacter pylori can in fact be killed by this fatty acid [16]. Another interesting observation is that capric and lauric acid are reported to inhibit COX-I and COX-II [17]. Stearic acid (18:0) does not seem to increase serum cholesterol concentration, and is not atherogenic [10,13].

It would appear, accordingly, that some of the saturated fatty acids in milk have neutral or even positive effects on health. In contrast to this, the saturated fatty acids lauric-, myristic-(14:0) and palmitic (16:0) acid have low-density lipoprotein (LDL)- and high-density lipoprotein- (HDL) cholesterol-increasing properties [13]. High intake of these acids raises blood cholesterol levels [13], and diets rich in saturated fat have been regarded to contribute to development of heart diseases, weight gain and obesity [4]. Association between consumption of milk and milk products and serum total cholesterol, LDL cholesterol and HDL cholesterol has been reported [18]. High cholesterol levels are a risk factor for coronary heart disease (CHD), with LDL cholesterol and a high ratio between LDL and HDL cholesterol enhancing the risk of CHD [19,20].

Several intervention studies have shown that diets containing low-fat dairy products have been associated with favourable changes in serum cholesterol [21-23]. However, milk fat consumption has been shown to have less pronounced effects on serum lipids than could be expected from the fat content [24,25]. To our knowledge epidemiological cohort studies does not show a higher risk for diseases in persons with high intakes of dairy fat, as also shown by Elwood et al. [26]; cohort studies provide no convincing evidence that milk is harmful. On the contrary, several studies have found a lack of association between milk consumption and CHD [27-30]. Two Swedish studies have shown that cardiovascular risk factors were negatively associated with intake of milk fat [31,32]. A Norwegian study suggests that intake of dairy fat or some other component of dairy products, as reflected by C15:0 as marker in adipose tissue may protect persons at increased risk from having a first myocardial infarction (MI), and that the causal effects may rely on other factors than serum cholesterol [33]. It has been shown that 34 grams dairy fat per day gives no negative effect on odds ratio for myocardial infarction [34]. As reported by Sjogren et al. [35], fatty acids typically found in milk products were associated with a more favourable LDL profile in healthy men (i.e., fewer small, dense LDL particles), and they concluded that men with high intakes of milk products had an apparently beneficial and reduced distribution of the harmful small, dense LDL particles [35].

A Canadian 13 year follow up study analysed plasma LDL sub fractions with different density, and showed that cardiovascular risk was largely related to accumulation of small, dense LDL particles [36]. The small, dense LDL particles are also reported to be associated with hypertriglyceridemia [37], insulin resistance [38], the metabolic syndrome and increased risk for CHD [39,40]. Saturated fatty acids increase the serum concentration of both LDL- and HDL cholesterol. In a metaanalysis of 60 selected trials Mensink et al. [13] reported that saturated fatty acids gave an unchanged ratio between total cholesterol and HDL cholesterol if carbohydrates replaced saturated fatty acids. It was shown by Hostmark et al. [41] that an index reflecting the LDL/HDL balance, ATH-index = (total cholesterol-HDL)*apoB/(apoA*HDL), improved the discrimination between controls and subjects with coronary artery stenosis. Unlike this, the distribution of total cholesterol was similar in controls and patients, as evaluated by coronary angiography. In keeping with these early results, in the INTERHEART case-control study on risk factors associated with myocardial infarction in 52 countries, an increase in apo B/apo A1 ratio was shown to be the strongest risk factor for myocardial infarction [5]. ApoB/apo A1 was found to be a stronger risk factor than total cholesterol alone, or ratio between LDL and HDL cholesterol (Yusuf, personal information).

Increased levels of C-reactive protein (CRP) have been associated with inflammation [42], and CRP is recognized as a risk factor for CHD and metabolic syndrome [42,43]. Fredrikson et al. [43] found, however, no significant association between CRP and intake of saturated fat. These studies are in agreement with others [44].

The increase in HDL cholesterol caused by the saturated fatty acids lauric-, myristic- and palmitic acid [13] has beneficial effects as the reverse cholesterol transport is increased [4]. HDL can also act as an antioxidant and prevent oxidation of LDL particles in the blood, and it may protect against infections and against toxins from microbes [45].

#### Unsaturated fatty acids

Oleic acid (18:1c9) is the single unsaturated fatty acid with the highest concentration in milk accounting to about 8 g/litre whole milk [9] (Table 1). Accordingly milk and milk products contribute substantially to the dietary intake of oleic acid in many countries. In Norway about a quarter of the average intake of oleic acid comes from milk and milk products [3]. Oleic acid is considered to be favourable for health, as diets with high amounts of monounsaturated fatty acid will lower both plasma cholesterol, LDL-cholesterol and triacylglycerol concentrations [46], and replacement of saturated fatty acids with cis-unsaturated fatty acids reduces risk for coronary artery disease [13]. Several studies also indicate a cancer protective effect of oleic acid, but the data are not fully convincing [47].

Fatty acids are the main building material of cell membranes. The unsaturated fatty acids are reactive as they may give oxidative stress with free radicals and secondary peroxidation products (different aldehydes such as malonedialdehyde and 4-hydroxynonenale) that may be harmful to proteins and DNA in the cells [48,49]. This may contribute to cancer [49] and to mitochondrial aging processes caused by mutations in mitochondrial DNA [50]. The enzyme lechitin/cholesterol acyl transferace (LCAT), having an important role in reverse cholesterol transport, is sensitive to oxidative stress [51] and it is also inhibited by minimally oxidized LDL [51]. Oleic acid is more stable to oxidation than the omega-3 and omega-6 fatty acids, and it can partly replace these fatty acids in both triacylglycerols and membranelipids. A high ratio between oleic acid and polyunsaturated fatty acids will protect lipids in i.e. LDL towards attack from oxidative stressors such as cigarette smoke, ozone and other oxidants. Studies have shown that a diet rich in monounsaturated/polyunsaturated fatty acids give better protection against atheromatosis and CVD than a diet rich in polyunsaturated fatty acids [52,53].

Milk fat is rich in oleic acid (about 25 % oleic acid) and it has a very high ratio oleic acid/polyunsaturated fatty acids. A diet rich in milk fat therefore may help to increase this ratio in the total dietary fatty acids. A high intake of meat from i.e. sheep may be expected to have similar effect. This might partly explain why mortality by cardiac disease has been lower in Iceland compared to the other Scandinavian countries [54], and the average age of living has been higher [55] despite of higher intake of saturated fat (coming from both mutton and milk).

The concentration of PUFA in milk is about 2 g/l [9], and the main PUFA in milk are linoleic- (18:2 omega-6) and alpha-linolenic (18:3 omega-3) acid (Table 1). These fatty acids may be converted to fatty acids with 20 carbon atoms, i.e. arachidonic acid (20:4 omega-6) and eicosapentaenoic acid, (EPA) (20:5 omega-3), and further converted to eicosanoids; metabolically very active compounds with local functions. Eicosanoids derived from linoleic acid, via arachidonic acid, may enhance blood platelet aggregation and thereby increase the coronary risk, in contrary to eicosanoids produced form the long omega-3 fatty acids [56]. EPA has the ability to partially block the conversion of the omega-6 fatty acids to harmful eicosanoids, thereby reducing the cardiovascular risk and inhibiting tumour genesis. PUFA may also affect signal transduction and gene expression [57,58]. It is conceivable therefore that the type of fatty acid in the membrane governs several metabolic functions.

It has been argued that the Mesolithic man had a ratio of 1–4:1 between the omega-6 and omega-3 fatty acids, against now in most European diets 10–14:1 [59]. Eskimos, and some populations in Japan, having a high intake of omega-3 fatty acids, also have a low rate of coronary heart diseases, and of some cancers [59]. Conceivably, protection against cardiovascular diseases and cancer would be related to the ratio of EPA plus oleic acid to omega-6 fatty acids in the diet, and hence in the body.

In milk the ratio between omega-6 and omega-3- fatty acids is low and favourable compared to most other non-marine products (Table 1). This ratio is greatly influenced by the feeding regime, and may with favourable feeding be as low as 2:1 (see later). Comparing the omega-6 to omega-3 ratio in milk in the Nordic countries, Thorsdottir et al. [60] has reported the lowest ratio in Iceland: 2.1:1, compared to 4.7:1 in milk from the other Nordic countries. It has been suggested that the higher supply of omega-3 fatty acids from milk in Iceland might explain the lower prevalence of type-2 diabetes and CHD mortality in Iceland compared to the other Nordic countries [60]. A Norwegian study showed reduced risk of premenopausal breast cancer with milk intake [61]. With proper feeding regime, milk and meat from ruminants can in fact be the main source of omega-3 fatty acids in the human diet, as is the case in France [62].

According to the above considerations a favourable meal should be rich in oleic acid, and have a low ratio between omega-6 fatty acids and omega-3 fatty acids, perhaps near 1–2:1. Indeed, milk fat fits into this description probably better that any other food item.

#### Conjugated linoleic acid (CLA)

Bovine milk, milk products and bovine meat are the main dietary sources of the cis9, trans 11 isomer of conjugated linoleic acid (9c,11t-CLA) [63]. In most cases this isomer is the most abundant CLA-isomer in bovine milk [64]. Minor amounts of other geometrical and positional isomers of CLA also occur in milk (such as the 7t, c9 and 10t, 12c-CLA), with different biological effects [65,66]. Milk content of 9c,11t-CLA vary considerably (see later), but may constitute about 0,6 % of the fat fraction [67,68].

The health effects of CLA have been discussed [69]. Administration of 9c,11t CLA has shown to modulate plasma lipid concentration in both human and animal models [70,71]. Some studies [70-72] but not all [73] have shown that addition of CLA isomer mixtures (9c,11t and 10t,12c) to a diet affects plasma lipids. Studies have shown that especially 9c,11t-CLA can improve plasma cholesterol status [70,71]. In a study with healthy men Tricon et al. [70] found a significant reduction in plasma total cholesterol concentration by 9c,11t-CLA. The results concerning the effects of CLA on serum triglycerides are controversial [66,70,74,75]. Tricon et al. [70] observed a decrease in serum triglycerides by 9c,11t-CLA compared to 10t,12c-CLA in humans, and Roche et al. found serum triglycerides and unesterified FA to be decreased by 9c,11t-CLA in ob/ob-mice [66].

In experimental animals CLA has been shown to have anticarcinogenic effects [76]. Prospective data from a Swedish study suggest that high intakes of high-fat dairy foods and CLA may reduce the risk of colorectal cancer [77]. The knowledge of CLA's effects in metabolism and the reported anti-proliferative and pro-apoptotic effect of CLA on various types of cancer cells [78] makes CLA to an interesting, and possible therapeutic agent in nutritional cancer therapy. The mechanisms by which CLA might affect metabolism are many. It is suggested that CLA competes with arachidonic acid in the cyclooxygenase reaction, resulting in reduced concentration of prostaglandins and tromboxanes in the 2-series [79]. CLA may suppress the gene expression of cyclooxygenase [80], and reduce the release of pro-inflammatory cytokines such as TNF-alpha and interleukines in animals [79]. CLA also activates the PPARs transcription factors [63], and CLA may reduce the initial step in NF-kappa B activation and thereby reduce cytokines, adhesions molecules and other stress-induced molecules [81].

#### Trans vaccenic acid (VA)

The main trans 18:1 isomer in milk fat is vaccenic acid, (18:1, 11t, VA), but trans double bounds in position 4 to 16 is also observed in low concentrations in milk fat [82].

The amount of VA in milk fat may vary; constituting 1.7% [83], or 4–6 % of the total fatty acid content [84]. Typically, the concentration of VA may be about 2–4% when the cows are on fresh pasture and about 1–2 % on indoor feeding [67]. Normally, naturally increase in 9c,11t-CLA in milk also results in increased concentration of VA [85].

VA has a double role in metabolism as it is both a trans fatty acid and a precursor for 9c,11t-CLA. As demonstrated by Kay et al. [86] approximately 90 % of 9c,11t-CLA in milk fat was produced endogenously involving delta-9-desaturation of VA. Vaccenic acid can be converted to 9c,11t-CLA in rodents [87], pigs [88] and humans [89].

Trans fatty acids have been shown to increase blood lipids [90]. Industrially produced trans fat are shown to increase the risk of coronary heart disease as they have adverse influence on the ratio of LDL on HDL, and on Lp(a) [44,91]. It has been questioned if VA has these same adverse effects. In one study with hamster, Meijer et al. [92] found that VA was more detrimental to cardiovascular risk than elaidic acid (18:1, 9t) due to a more increasing effect on LDL/HDL cholesterol ratio. Furthermore, Clifton et al. [93] showed that VA was an independent predictor of a first myocardial infarction. In contrast to this, it has been shown by Willett et al. [28] that trans fat from animals did not give an increased risk for CHD. As recently demonstrated by Tricon et al [85], a combination of naturally increased concentration of VA and 9c,11t-CLA in milk fat did not result in detrimental effects on most cardiovascular disease risk parameters. However, it remains to clarify if VA has unhealthy effects on blood lipids.

#### Phospholipids and glycosphingolipids

Phospholipids and glycosphingolipids accounts to about 1% of total milk lipids [8]. These lipids contain relatively larger quantities of polyunsaturated fatty acids than the triacylglycerols. They have functional roles in a number of reactions, such as binding cations, help to stabilize emulsions, affect enzymes on the globule surface, cell-cell interactions, differentiation, proliferation, immune recognition, transmembrane signalling and as receptors for certain hormones and growth factors [6]. Gangliosides are one of these components found in milk. Gangliosides (with more than one sialic acid moiety) are mainly found in nerve tissues, and they have been demonstrated to play important roles in neonatal brain development, receptor functions, allergies, for bacterial toxins etc [94].

### Protein

Bovine milk contains about 32 g protein/l [9] (Table 1). The milk protein has a high biological value, and milk is therefore a good source for essential amino acids. In addition, milk contains a wide array of proteins with biological activities ranging from antimicrobial ones to those facilitating absorption of nutrients, as well as acting as growth factors, hormones, enzymes, antibodies and immune stimulants [95,96]. The nitrogen in milk is distributed among caseins, whey proteins and non-protein nitrogen. The casein content of milk represents about 80% of milk proteins. Caseins biological function is to carry calcium and phosphate and to form a clot in the stomach for efficient digestion. The milk whey proteins are globular proteins that are more water soluble than caseins, and the principle fractions are beta-lactoglobin, alpha-lactalbumin, bovine serum albumin and immunoglobulins. Whey is the liquid remaining after milk has been curdled to produce cheese, and it is used in many products for human consumption, such as ricotta and brown cheese, and concentrated whey is an additive to several products e.g. bread, crackers, pastry and animal feed. The rate at which the amino acids are released during digestion and absorbed into the circulation may differ among the milk proteins, and whey proteins are considered as rapid digested protein that gives high concentrations of amino acids in postprandial plasma [97]. The benefit of drinking whey has been known for centuries, and two ancient proverbs from the Italian city of Florence say, "If you want to live a healthy and active life, drink whey" and, "If everyone was raised on whey, doctors would be bankrupt" [98].

Some of the milk proteins (e.g. secretory immunoglobulin A, lactoferrin, 1-antitrypsin, β-casein and lactalbumin) may be relatively resistant to digestive enzymes, and the whole protein or peptides derived from it, may exert their function in the small intestine before being fully digested [99].

As several bioactive proteins and peptides derived from milk proteins are potential modulators of various regulatory processes in the body, some of these are produced on an industrial scale, and are considered for application as ingredients in both 'functional foods' and pharmaceutical preparations. Although the physiological significance of several of these substances is not yet fully understood, both the mineral binding and cytomodulatory peptides derived from bovine milk proteins are now claimed to be health enhancing components that can be used to reduce the risk of disease or to enhance a certain physiological function [100]. Milk protein composition may differ among breeds [101]. For example the concentration of beta-casein A1 is low in milk from cows in Iceland and in New Zealand. It has been speculated that this proteins may have a role in the development of diabetes and cardiac disease [102]. However, later it was concluded in a review article that there is no convincing evidence that the A1 beta-casein of cow milk has any adverse effect in humans [103].

#### Milk peptides and blood pressure

Several studies has suggested that there is an association between milk consumption and blood pressure; as hypertension is inversely related to milk consumption in some epidemiological- and intervention studies [104]. It has been suggested that some milk peptides have antihypertensive effects, both by inhibiting angiotensin-converting enzyme, having opoid-like activities, antithrombotic properties and by binding minerals [104].

#### Branched chain amino acids and other amino acids

Milk is especially rich in essential amino acids and branched chain amino acids. There is evidence that these amino acids have unique roles in human metabolism; in addition to provide substrates for protein synthesis, suppress protein catabolism and serve as substrates for gluconeogenesis, they also trigger muscle protein synthesis and promote protein synthesis [105,106]. Essential amino acids are shown to be more important than non-essential amino acids in muscle protein synthesis [107], and the branched chain amino acid leucine in particular triggers muscle protein synthesis which is sensed by the insulin-signalling pathway [106]. The stimulated insulin secretion caused by milk, is suggested to be caused by milk proteins, and as shown by Nilsson et al. [97] a mixture of leucine, isoleucine, valine, lysine and threonine resulted in glycemic and insulinemic response resembling the response seen after ingestion of whey. A combination of milk with a meal with high glycaemic load (rapidly digested and absorbed carbohydrates) may stimulate insulin release and reduce the postprandial blood glucose concentration [108]. A reduction in postprandial blood glucose is favourable, and it is epidemiological evidence suggesting that milk may lower risk of diseases related to insulin resistance syndrome [109].

#### Taurine

The concentration of taurine is high in breast milk (about 18 mg/l) and in colostrum from cow, but in regular bovine milk it is not high; about 1 mg/l [110]. Goat milk is however very rich in taurine: 46–91 mg/l [110]. Taurine is an essential amino acid for preterm neonates, and specific groups of individuals are at risk for taurine deficiency and may benefit from supplementation, e.g. patients requiring long-term parenteral nutrition (including premature and newborn infants); diabetes patients, those with chronic hepatic, heart or renal failure [111,112]. It is suggested that during parenteral nutrition, supplementation of 50 mg taurine per kg body weight may be required [113].

Taurine is the most abundant intracellular amino acid in humans. It may be synthesized in the body from methionine and cysteine, but in healthy individuals the diet is the usual source of taurine. It is implicated in numerous biological and physiological functions: bile acid conjugation and cholestasis prevention, antiarrhythmic/inotropic/chronotropic effects, central nervous system neuromodulation, retinal development and function, endocrine/metabolic effects and antioxidant/anti-inflammatory properties [111]. Taurine has been shown to have endothelial protective effects [114], it may function principally as a negative feedback regulator, helping to dampen immunological reactions before they cause too much damage to host tissues or to the leukocytes themselves [115], and it is shown to be analgesic [112,116].

#### Glutathione (GSH)

Fresh milk may be a good source of glutathione, a tripeptide of the sulphur amino acid cysteine, plus glycine and glutamic acid. In the organism glutathione has the role as an antioxidant. Glutathione can be oxidized forming GSSG (oxidized glutathione), and in this reaction it may remove reactive oxygenspecies (ROS), thereby regulating the level of ROS in the cells. Glutathione participates in regulation of insulin production in the pancreatic cells, as ROS inhibit expression of the pro insulin gene. Glutathione appears to have different important roles in leukocytes, as a growth factor, as an anti-apoptotic factor in leukocytes and to regulate the pattern of cytokine secretion [117]. GSH, moreover, is also central for antioxidative defence in the lungs, which may be very important in connection with lower respiratory infections including influenza [118].

## Minerals, vitamins and antioxidants

Milk contains many minerals, vitamins and antioxidants. The antioxidants have a role in prevention of oxidation of the milk, and they may also have protective effects in the milk-producing cell, and for the udder. Most important antioxidants in milk are the mineral selenium and the vitamins E and A. As there are many compounds that may have antioxidative function in milk, measurement of total antioxidative capacity of milk may be a useful tool [119].

### Calcium

The calcium concentration in bovine milk is about 1 g/l (Table 1). Dairy products provide more than half of the calcium in the typical American diet [4], and daily intake of milk and milk products thus has a central role in securing calcium intake. In human nutrition adequate calcium intake is essential. Getting enough calcium in the diet gives healthy bones and teeth, and it may also help prevent hypertension, decrease the odds of getting colon or breast cancer, improve weight control and reduce the risk of developing kidney stones [4].

### Selenium

The selenium concentration in body fluids and tissues are directly related to selenium intake. The selenium concentration in Scandinavian food is low, and the concentration in Norwegian bovine milk is about 11 ug/l (own results, 2006), and 37 ug/l in the US [9]. For plant products the situation is even worse; the selenium concentration in wheat flour (whole grain) is less than 20 ug/kg in Norwegian wheat (own results), compared to 707 ug/kg in the US [9].

Selenium is important in human health; it has a role in the immune- and antioxidant system and in DNA synthesis and DNA repair [120]. Selenoprotein P is an antioxidative defence enzyme having similar function as the selenoenzyme phospholipid hydro peroxide glutathione peroxidase (Gpx-4) inside the cells and it also protects LDL towards peroxidation [121,122]. A strong negative correlation between the concentration of selenium and the concentration of plasma lipid peroxidation products has been reported in a Canadian study [123]. These observation are in line with epidemiological observations from USA, where a strong negative correlation between mortality of ischemic cardiac disease and hypertension among men and women in the age group 55–64 years comparing states with different selenium intake [124,125].

Selenium protects against many (but not all) types of cancer [4]. There are indications that selenium may protect against asthma, and that low selenium intake may worsen the asthma symptoms [126]. Selenium deficiency has even been linked to adverse mood states [127]. Selenium is also a component of enzymes involved in metabolism of thyroid hormone.

As selenium is of fundamental importance to human health, the low selenium availability in Scandinavian soil is of concern. Different strategies can be used to increase human selenium intake, and addition of selenium-rich yeast to the feed of domestic animals is one option. Recommended daily intake of selenium is 55 ug [4], and the optimal selenium concentration in bovine milk may be discussed. If milk contains about 50–100 ug selenium/l, it would be a good selenium source.

### Iodine

Iodine is an essential component of the thyroid hormones. These hormones control the regulation of body metabolic rate, temperature regulation, reproduction and growth.

The recommended iodine intake is 150 ug/d for adults [4]. Accordingly, a daily intake of 0.5 litres milk with an average content of 160 ug iodine/l meets about 50% of the requirement (Table 1). However, it is important to underline the great seasonal variation in iodine content of milk (see later).

### Magnesium

Magnesium is ubiquitous in foods, and milk is a good source, containing about 100 mg/l milk [9]. Recommended intake is 400 mg/day for men and 310 mg/day for women [4]. Magnesium has many functions in the body, participating in more than 300 reactions. Magnesium deficiency has been linked to atherosclerosis, as studies have shown that deficiency may give oxidative stress [128]. Magnesium may also have a role in reducing asthma, and experimental studies of persons with asthma suggest that magnesium infusion may have a place in the acute treatment of asthma [129]. A possible mechanism may be that magnesium together with taurine dampens the signaling effects of a too high calcium release inside the cells [111,130]. Magnesium deficiency may occur following kidney disease and after use of some diuretic drugs. Magnesium deficiency in elderly is observed, and may be a result of poor appetite or unbalanced diet.

### Zinc

Zinc is an essential part of several enzymes and metalloproteins. Zinc has several functions in the body, in DNA repair, cell growth and replication, gene expression, protein and lipid metabolism, immune function, hormone activity, etc [4]. Milk is a good zinc source; containing about 4 mg/l [9]. Recommended intake is 8 and 11 mg/day for adult female and male [4]. The bioavailability of zinc is better from milk than from vegetable food [4], and inclusion of milk in the diet may improve total bioavailability of zinc [131].

### Vitamin E

Vitamin E concentration in milk is about 0,6 mg/l [9] (Table 1), but may increase 3–4 folds by proper feeding regimes (see later). Recommended intake is 15 mg/day [4]. Vitamin E is not a single compound; it includes tocoferols and tocotrienols. In whole milk, alpha-tocopherol is the major form of vitamin E (>85%); gamma-tocopherol and alpha-tocotrienol are present to a lesser extent, about 4 % each of the sum of tocoferols and tocotrienols [132]. Observational studies indicate that high dietary intake of vitamin E are associated with decreased risk for cancer and coronary heart disease, and that vitamin E can stimulate T-cells and increase the immune defence system. Milk seems to be a food item favouring absorption and transportation of vitamin E from ingested food into the chylomicrons [133].

### Vitamin A

Milk is a good source of retinoids, containing 280 ug/l [9] (Table 1). Recommended daily intake is 700–900 ug/day [4]. Vitamin A has a role in vision, proper growth, reproduction, and immunity, cell differentiation, in maintaining healthy bones as well as skin and mucosal membranes [4].

### Folate

Bovine milk contains 50 ug folate/l [9]. Studies indicate that 5-methyl-tetrahydrofolate is the major folate form in milk [134]. Recommended intake of folate is 400 ug/day for adults [4]. Many scientists believe that folate deficiency is the most prevalent of all vitamin deficiencies [4]. It is generally accepted that folate supplementation (400 ug/day) before conception and during the first weeks of pregnancy reduces the risk of neural tube defects. A recent study has shown that higher total folate intake was associated with a decreased risk of incident hypertension, particularly in younger women [135]. In addition, folates may have a protective role to play against coronary heart disease and certain forms of cancer, but sufficient evidence is not yet available [136]. The complexity of the folate metabolism suggest that different metabolites of folate are involved in different reactions, and that dihydrofolate and 5-methyl-tetrahydrofolate are the active compounds in growth-inhibition in colon cancer cells [137].

The bioavailability of folate varies [138]. Folate-binding proteins occur in unprocessed milk, pasteurised milk, spray-dried skim milk powder and whey [134]. Animal and human studies have suggested that these components enhance food folate bioavailability, and it is shown that inclusion of cow milk in the diet enhanced the bioavailability of food folate [139]. In a population-based study, the consumption of milk and yogurt were inversely associated with serum total homocysteine concentrations, and the authors explained this association by intakes of folate and riboflavin [140].

### Riboflavin

Milk is a good source of riboflavin, 1.83 mg riboflavin/l milk (Table 1). Daily recommended intake is 1.1 and 1.3 mg for women and men, respectively [4]. Riboflavin is part of two important coenzymes participating in a numerous metabolic pathways in the cell. It has a role in the antioxidant performance of glutathione peroxidase and DNA repair via the ribonucleotid reductase pathway.

### Vitamin B12

Milk is also a good source of vitamin B_12_, being 4.4 ug/l [9]. The daily recommendation is 2.4 μg [4]. Vitamin B_12 _is found only in animal foods, and plays a central role in folate and homocysteine metabolism, by transferring methyl groups. Vitamin B_12 _deficiency may cause megaloblastic anaemia and breakdown of the myelin sheath.

## Bacterial flora of milk

Milk samples from normal healthy mammary glands contain many strains of bacteria [141]. To prevent diseases caused by pathogenic bacteria in milk and to lengthen the shelf life of milk, treatment such as cooling and pasteurization or membrane filtration is needed. To preserve milk, addition of selective, well-documented strains of starter cultures for fermentation is a method that has been used for centuries.

## Fermented milk

Historically, the seasonal variation in milk production made it necessary to preserve milk. The Nordic countries including Iceland have a long tradition for using fermented milk, and the consumption of fermented milk is about 20 kg per person [2].

During fermentation bacteria and yeasts convert lactose in the milk to various degradation products depending on the species present. Lactobacilli and streptococci give rice to lactic acid and monosaccarides (especially galactose). Bifidobacteria give rice to lactic acid, acetic acid and monosaccarides, while yeasts, present only in some few fermented milk products, produce CO_2 _and ethanol [2]. Different bacterias may be used for fermentation, giving products of special flavour and aroma, and with several potential health beneficial metabolites [142]. The bacteria contain cell wall components that bind Toll-like receptors on dendritic cells (and also other leucocytes) found in the mucosa of the small intestine and colon, thus stimulating the Th1 immune response [143]. It has been shown that fermented milk stimulates the Th1 immune response, and down-regulates the Th2 immune response [144]. The immune system may thus be strengthened against cancer, virus infections and allergy [145]. Bacterial DNA has also a similar effect, binding to Toll-like receptor-9 [146]. Some bacteria can also improve the intestinal microbial balance, and the fermented milk may have positive health effects both in the digestive channel and in metabolism. During the fermentation of milk, lactic acid and other organic acids are produced and these increase the absorption of iron. If fermented milk is consumed at mealtimes, these acids are likely to have a positive effect on the absorption of iron from other foods [147]. Lactic acid is also a poorer substrate for growth of pathogenic bacteria than glucose and lactose [148].

The low pH in fermented milk may also delay the gastric emptying from the stomach into the small intestine and thereby increase the gastrointestinal transit time [149]. Also, full-fat milk has been shown to increase the mean gastric emptying half-time compared to half-skimmed milk [150], and accordingly it might be favourable to gastric emptying and thus may have an effect on appetite regulation [150,151].

## Intolerance to milk components

The public "belief" that milk causes an inflammatory process and an increase in mucus production has not been confirmed [152,153]. It has been shown that respiratory symptoms was not associated with milk intake [152], and concluded that consumption of milk does not seem to exacerbate the symptoms of asthma, but in a few cases people with cow's milk allergy may have asthma-like symptoms after milk consumption [153]. However in cells from another tissue; mucin producing cells of gastric mucosa, alpha-lactalbumin stimulates mucin synthesis and secretion [154].

### Milk allergy

Most milk proteins, even proteins present at low concentrations, are potential allergens. A person may be allergic to casein or whey proteins or to both. Milk allergy may arise in small children (0–3 years) and it is estimated that 2–5% of the children has milk allergy [155]. After the age of three years it is no longer a problem for most children.

Milk allergy reactions may either be of the type 'rapid onset' or the 'slower-onset' type. The rapid type comes suddenly with symptoms of e.g. wheezing, vomiting, anaphylaxis. The slower-onset reactions are more common and symptoms develop over a period of hours or days after ingesting milk, and may include loose stool, vomiting, fussiness, reduced weight gain etc. As these symptoms are more general, this type is difficult to diagnose.

Cow's milk allergy may be treated by completely avoiding milk proteins. Epitopes on milk proteins have been shown to be both conformational and linear epitopes, widely spread throughout the protein molecules. Due to the great variability and heterogeneity of the human IgE response, no single allergen or particular structure has been found to be a major part of milk allergenicity [156].

An interesting study from Germany showed that the children of farmers had less allergy, in spite of the fact that these children were drinking more whole- milk than other children not living on farms[157].

### Intolerance to milk proteins

There has been speculation if milk proteins may have a role in Attention Deficit Hyperactivity Disorder (ADHD), autism, depressions and schizophrenia in some cases. There are major supports to the hypothesis that ADHD may be linked to increased levels of neuroactive peptides and increased urinary peptide levels [158,159]. A diet free of milk, milk products and gluten may in many cases give reduced ADHD symptoms [158]. Further, opioid peptides derived from food proteins (exorphins) have been found in urine of autistic patients [160]. This area of investigation is important and large scale, good quality randomised controlled trials are needed.

### Lactose intolerance

The lactose concentration in bovine milk is about 53 g/l [9]. People often confuse a milk allergy with lactose intolerance, but they are not the same thing. Lactose intolerance is common in many adults throughout the world, and is caused by deficiency of intestinal lactase (hypolactasia). Lactose maldigestion occurs in about 75 % of the worldwide population and about 25 % of the US population [4]. In Scandinavian counties it varies between 2% and 18% [4]. Avoiding all lactose is seldom necessary, and persons with hypolactasia can usually ingest limited amounts of milk without having annoying symptoms. Individual differences in gut micro flora may be one reason for large variations in amounts of milk that is tolerated. To ingest milk with a meal may also improve tolerance. Instead of drinking regular milk, fermented milk may be an option, because fermented milk contains less lactose than fresh milk, and that it also may contain bacterial lactase that may be activated when the fermented milk reaches the gut [161].

### Galactosemia

Digestion of lactose in the intestine, and fermentation of milk gives increased concentrations of galactose. Galactose is catabolized by the Leloir pathway by phosphorylation at position 1, and then converted to UDP-galactose and glucose-1-phosphate [162]. Defects in enzymes in this pathway may result in galactosemia in humans, and early onset cataract. In young women ovarian failure at a very early age has been observed following galactose accumulation. Cramer et al. [163] studied the relation between age-specific fertility rates, the prevalence of adult hypolactasia and per capita milk consumption. They found that fertility at high ages is lower with high per capita consumption of milk and greater ability to digest its lactose component. These demographic data thus add to existing evidence that dietary galactose may deleteriously affect ovarian function.

The level of galactose in fermented milk products depends on growth conditions of the different organisms and fermentation time, and for example after 24 h fermentation, the concentration of galactose has been reported to about 20 g/litre [164]. A study in rats showed that administration of galactose in the form of lactose seemed to be less toxic than when galactose was fed [165]. High levels of galactose as well as glucose may cause glycation of proteins, form advanced glycation end products, and the activation of polyol metabolism. This may accelerate generation of reactive oxygen species (ROS) and increases in oxidative chemical modification of lipids, DNA, and proteins in various tissues.

## Possible concerns of milk in current use

Within modern societies the milk has to be treated in different ways to keep for several days. This processing includes steps that may be of concern. In fresh milk each lipid globule is surrounded by apical plasma membrane from the mammary epithelial cell. It is not known whether the milk homogenisation, when the fat globules with their globule membrane are broken up into many new small lipid droplets with just a small fragment of the originating membrane, might have health implications.

Proteins and peptides are heat sensitive, and their bioactivity may be reduced by pasteurisation of milk. Heating of milk may also result in the formation of potentially harmful new products i.e. when carbohydrates in milk react with proteins [166]. Also the amount of some vitamins and antioxidants may be reduced by heating. Glutathione may easily be destroyed during storage [167]. The glutathione concentration in human breast milk was reduced by 81, 79 and 73 % by storage at either -20 degrees C, 4 degrees C or at room temperature for 2 h, respectively [167]. To treat milk in a way that preserves the vitamins, proteins and peptides is therefore an important task and a challenge for the dairy industry. Some dairies now membrane filtrate the milk in stead of pasteurisation, and application of non-thermal processing technologies may give health benefits.

## Further improvements of the nutritional quality of bovine milk

Several components in bovine milk which are of great importance in human nutrition may be significantly altered by the feeding regime [168]. The principal effects of feeding on milk content of these components are summarized and briefly discussed below.

### Fat content and composition

The fatty acids of bovine milk are derived from two sources. The first source is fatty acids supplied to the udder by the blood, composed of fatty acids absorbed from the intestine and mobilized from the adipose fat tissue, mainly palmitic acid (16:0), stearic acid (18:0) and longer chained fatty acids. The second source is derived from circulating blood acetate and butyrate produced during fermentation in the rumen (*de novo synthesis)*, and fatty acids up till 14 carbon atoms are synthesised in the udder. Palmitic acid in milk originates from both *de novo synthesis *and from circulating blood. Due to the extensively biohydrogenation of dietary unsaturated fatty acids in the rumen, the supply of these fatty acids to the udder is low. However, in the udder desaturation of fatty acids like 12:0, 14:0, 16:0 and 18:0 take place, and the products being 12:1, 14:1, 16:1 and 18:1, respectively. The preferred substrate for the desaturating enzyme; delta-9-desaturase, is stearic acid. Therefore is bovine milk a relatively good source of oleic acid (18:1, cis 9). The udder enzymes can not make double bonds in omega-3 and omega-6 positions. Consequently, milk content of linoleic acid and alpha-linolenic acid depends on the supply of these to the udder.

Conjugated linoleic acid (9c,11t-CLA) in milk originates from two sources. A small part originates from incomplete biohydrogenation of linoleic acid in the rumen which are absorbed from the small intestine transported to the udder and included in the fat synthesis. Most of the 9c,11t-CLA originates, however, from vaccenic acid which is an intermediate from biohydrogenation of unsaturated fatty acids in the rumen. After absorption and transportation by the blood to the udder, a portion of the vaccenic acid is desaturated by delta-9-desaturase to CLA. There is a close positive correlation between milk content of vaccenic acid and 9c,11t-CLA [86,169].

The effect of feed on milk fat content and fatty acid composition is comprehensively discussed [68,170-172]. There are large variations in fat synthesis in the udder, and the fat content and fatty acid composition are the most modifiable of the main components in milk. Some feeding strategies to obtain milk with altered fatty acid composition are summarized in Table 2. There are seasonal variations for the major fatty acids [67,82]. Milk from grazing dairy cows contain significantly higher proportion of oleic acid than milk produced on traditional indoor feeding composed of concentrates and conserved roughages [67]. Typically, CLA content in milk produced on pasture is at least twice of that obtained by indoor feeding [67,82]. Moreover, the proportion of alpha-linolenic acid increases more than linoleic acid, resulting in a lower ratio between omega-6 and omega-3 fatty acids. Milk fat from cows fed an in-door diet consisting of conserved grass and concentrate have a ratio between omega-6 and omega-3 fatty acids of about 4:1 [67,82], but in summer when the cows are out on pasture and have a high intake of grass the ratio may be reduced to about 2:1 [8,67,82,172]. These positive effects of pasture on the fatty acid composition of milk are mainly attributed to the high content of polyunsaturated fatty acids, especially alpha-linolenic acid, in grasses at early stage of maturity [173].

**Table 2:** Components in bovine milk and their chances to be modified according to feeding strategies, substrates involved in their synthesis and feeding strategies that may be used

**Milk component**	**Possibility for modification**^1^	**Substrates involved in synthesis**	**Feeding strategies**
Fat	Moderate	↓Acetic-and butyric acid for de novo synthesis	High intake and proportion of concentrates
		↓Long unsaturated FA	High intake and proportion of unsaturated fatty acids
Saturated fatty acids	Minor to moderate	↓Acetic-and butyric acid for de novo synthesis.	Low intake and proportion of roughages
		↓Long saturated FA	Low proportion saturated/unsaturated fatty acids
Oleic acid	Moderate	↑Oleic acid	High intake/proportion of oilic acid
		↑Stearic acid	High intake/proportion of stearic acid
		↑Stearic acid	High intake/proportion of polyunsaturated C18-acids Pasture
CLA	Considerable	↑CLA	High intake/proportion of linoleic acid.
		↑Vaccenic acid	High intake/proportion of unsaturated FA. Pasture
Vaccenic acid	Considerable	↓Vaccenic acid	Low intake of oleic- and linolenic acid
Ratio omega 6/3	Moderate	↓Omega 6:3 FA	High intake/proportion of linolenic acid. Pasture
Protein	Minor	↑Energy supply	High intake of diet with relatively low content of fat, but high energy concentration
		↑Amino acid supply	Favourable conditions for microbial protein synthesis in the rumen. High intake/proportion of dietary by-pass protein
Calcium	Minor		
Magnesium	Minor		
Zinc	Minor		
Iodine	Considerable		Supplementation
Selenium	Considerable		Supplementation
Vitamin E	Considerable	Vitamin E	Pasture, well preserved silages, concentrates with naturally high content, mineral supplementation
Carotene/Vitamin A	Considerable	Carotene/Vitamin A	Pasture, well preserved silages, concentrates with naturally high content, mineral supplementation
Folate	Minor		
Riboflavin	Minor		
Vitamin B_12_	Minor		

^1^Minor; ≈ < 25 % change. Moderate; ≈ 25–100 % change. Considerable; ≈ > 100 % change.

### Protein content and composition

In general, milk protein content is relatively unresponsive to feeding factors. However, under most conditions, energy-, but also protein supply, is feed related factors with most pronounced effect on milk protein content (Table 2). Milk protein content may be negatively influenced by high intake of dietary fat by the lactating cow [168]. Thus, there may be a conflict between milk fatty acid composition and protein content. The feeding regime has only small impact on the proportion of the different types of milk proteins and consequently the amino acid composition [101], and will therefore not be further discussed in this review. However, heat treatment of dairy products leads to structural changes of proteins and main whey proteins are modified to lactulosyl residues [174].

### Content of minerals

Bovine milk contain a vide range of minerals [4]. Milk concentrations of some minerals that are of special importance in human nutrition are given in Table 2. The calcium concentration in milk is relatively constant, with some variations throughout lactation. Most of the calcium is in the aqueous compartment and it is primarly (65%) associated with casein [175]. The calcium concentration in milk is relatively constant because milk content of casein is unresponsive to feeding factors. Milk content of magnesium and zinc also show only small variations.

The concentration of selenium in bovine milk is related to selenium concentration in the feed, and there are great variations worldwide. In South Dakota the selenium concentration in milk is reported to be between 160 and 1300 ug/l, whereas the concentrations in milk from low-selenium regions may be from 5 to 30 ug/l [176], as in Scandinavia and northern Europe. A Swedish study showed that the average selenium concentration in milk was 14 ug/l and the concentration was more than doubled after supplementation with 3 mg selenium daily from selenium-enriched yeast [177].

As much as about 25 percent of the iodine intake may be excreted in milk [178]. Therefore, milk content of iodine also varies depending on the iodine content and availability in the feeds used. A study on milk and milk products in Norway [179], showed that milk from the summer season had significantly lower iodine concentration (88 ug/l) compared with milk from the winter season (232 ug/l). This is explained by the use of more supplementary feeds enriched with iodine during the winter season. Dairy products supply much of the dietary intake of iodine; in Norway the most [179], and in US the second most [4] of the iodine intake.

### Content of vitamins

Vitamins are not synthesized in the udder. Milk content of the fat-soluble vitamins A and E reflects their content in the feed (Table 2). In general, the content of these vitamins in feed plants decrease with maturity and are higher in fresh than conserved material. There are therefore regional and seasonal variations in these vitamins due to feeding regimes [180], with the highest concentrations in fresh grasses at early stage of maturity. For example a study in Finland shows that the concentration of vitamin E in milk is 3–4 times higher during summer than in winter [181]. Enriching the supplementary feeds with proper sources of these vitamins may increase the milk content during the winter season.

All vitamins of the B-complex (riboflavin and vitamin B_12_, in Table 2) are synthesized by the rumen microbes, normally in sufficient amounts to cover the animal's needs. Milk content of B-vitamins are, however, relatively unrelated to their intake because the amount synthesized by the rumen microbes are unregulated according to the amount ingested [182].

## Summing up: Improvements of the nutritional quality of bovine milk

Different countries have different health challenges. In Norway the following modifications of milk composition may be most relevant:

• Secure a low omega-6 to omega-3 ratio, close to 2/1.

• Increase the proportion of oleic acid to 25%–30% of milk fat at the expense of palmitic acid.

• Have knowledge of how to increase the concentration of 9c,11t-CLA,

• Secure a low proportion of vaccenic acid

• Increase the selenium concentration in milk

• Secure a constant content of iodine.

## Conclusion

Consumption of 0.5 litre milk daily supplies a significant amount of many of the nutrients that are required daily. Milk components take part in metabolism in several ways; by providing essential amino acids, vitamins, minerals and fatty acids, or by affecting absorption of nutrients. Milk fat is diverse with a wide-ranging spectrum of fatty acids and lipids. Milk fat has been notified for decades, but as discussed in this review a moderate intake of milk fat has no negative health effects, on the contrary, many milk fat component have important roles in the body. Milk protein is especially rich in amino acids that stimulates muscle synthesis, and some proteins and peptides in milk have positive health effect e.g. on blood pressure, inflammation, oxidation and tissue development. Fermented milk has special health-promoting properties, e.g. stimulation of immune response and protection against cancer, virus and allergy, and fermented milk and full-fat milk may also delay gastric emptying from the stomach and possibly have an effect on appetite regulation. For some individuals, milk proteins, fat or milk sugar may cause health problems. Heat treatment of milk may also result in reduction of bioactive compounds and formation of potentially harmful products of carbohydrates and proteins. Milk can be significantly altered by changing the feeding regimes. Content of several fatty acids such as c9, t11-CLA and the ratio between omega-6 and omega-3 fatty acids are affected by the amount of grass and supplemental feeds (concentrate) in the diet. Milk content of several vitamins and minerals are also influenced by the cow's diet. Iodine and selenium are examples of trace elements that may be added to the feed, and thereby milk can be a good food source of these elements.

## Main conclusions

• Milk contains many important nutrients.

• A moderate intake of milk fat has no negative health effects.

• The increasing consumption of milk products added sugar and sugar containing jams should be questioned.

• It is possible to adjust feeding regimes to develop milk with increased content of healthy components such as selenium, iodine and some fatty acids.
